# Description of postoperative complications and bacterial contamination of wound soaker catheters used to administer postoperative local analgesia after mastectomy in 11 dogs: case series

**DOI:** 10.1007/s11259-024-10377-1

**Published:** 2024-04-24

**Authors:** María Suárez-Redondo, Manuel Fuertes-Recuero, Alba Guzmán-Soltero, Delia Aguado, María del Carmen Martín-Espada, Jorge Espinel-Rupérez, Gustavo Ortiz-Diez

**Affiliations:** 1 Complutense Veterinary Teaching Hospital, Complutense University of Madrid, Avda. Puerta de Hierro s/n, Madrid, 28040 Spain; 2 Department of Physiology, College of Veterinary Medicine, Complutense University of Madrid, Avda. Puerta de Hierro s/n, Madrid, 28040 Spain; 3 Department of Animal Medicine and Surgery, College of Veterinary Medicine, Complutense University of Madrid, Avda. Puerta de Hierro s/n, Madrid, 28040 Spain; 4 Department of Animal Health, College of Veterinary Medicine, Complutense University of Madrid, Avda. Puerta de Hierro s/n, Madrid, 28040 Spain; 5 School of Veterinary Medicine, Murdoch University, Perth, WA 6150 Australia

**Keywords:** Local anesthetic, Wound soaker catheters, Mastectomy, Complications, Bacterial contamination

## Abstract

Mastectomy is a common and painful procedure in dogs. Wound soaker catheters (WSC) are frequently used to reduce postoperative pain, including pain after mastectomy. The objectives of this case series were to describe the use of WSC for owner administration of postoperative local analgesia in dogs with mammary tumors treated surgically, to identify complications associated with WSC and to determine the frequency of bacterial colonization of the catheters. Twelve WSC were placed in 11 dogs during mastectomy surgery, left in place for three days, protected by a dressing and successfully managed by owners at home. No postoperative antibiotics were administered. No complications were identified in any cases. No bacterial growth was identified on bacteriological analysis of the twelve WSC. These results suggest that the use of WSC is a safe alternative for postoperative analgesia administration following mastectomy in dogs. Future studies comparing dogs with or without WSC with a larger number of dogs are needed to further evaluate efficacy and complications.

**Supplementary Information:**

The online version contains supplementary material available at 10.1007/s11259-024-10377-1.

## Introduction

Canine mammary tumours are the most common neoplasms in female dogs and surgical resection, such as regional or radical mastectomy, is the treatment of choice in most (Rasotto et al. 2017). Described postoperative complications following mastectomy include inflammation, hemorrhage, seroma, infection, dehiscence, and postoperative pain (Horta et al. 2015; Evans et al. 2021; Spare et al. 2021). Acute postoperative pain has been associated with a broad range of negative consequences, including prolonged recovery time, decreased food intake, increased medical costs, and increased morbidity and mortality (Gan 2017). Therefore, it is essential to provide effective analgesia during the postoperative period to improve the clinical outcome of patients (Bonnet and Marret 2005).

In many cases, drugs from different analgesic classes are used in combination as part of a multimodal plan.”(Bonnet and Marret 2005). Subcutaneous infiltration with continuous local anesthetic administration through wound soaker catheters (WSC) as a part of the postoperative multimodal analgesic plan has been described in human and veterinary medicine. In small animals, WSC have been reported to control postoperative pain following limb amputation (Abelson et al. 2009; Raske et al. 2015), total ear canal ablation (Radlinsky et al. 2005; Wolfe et al. 2006), and muscle dissection in dogs (Hardie et al. 2011), and fibrosarcoma resection in cats (Davis et al. 2007; Kazmir-Lysak et al. 2023). Despite their potential benefits, WSC have only been described in 3 dogs following mastectomy procedures (Moreno Velásquez et al. 2019; Evans et al. 2021). Lidocaine and bupivacaine are the most commonly administered drugs delivered through WSC (Abelson et al. 2009).

Described complications associated with WSC include: disconnection of the continuous infusion catheter (Radlinsky et al. 2005), premature catheter removal by the patient (Wolfe et al. 2006), catheter occlusion (Wolfe et al. 2006), seroma (Wolfe et al. 2006), surgical site infection (SSI) (Abelson et al. 2009) and suspected lidocaine neurotoxicity (Abelson et al. 2009). Potential complications associated with other wound catheters (closed drains) include increased wound drainage, delayed wound healing and increased SSI risk (Lu and Wright 2023). Therefore, similar complications could be expected with the use of WSC. However, none of these complications have been described associated with WSC in human or veterinary medicine. Moreover, no association between WSC and increased SSI risk has been described in veterinary medicine (Abelson et al. 2009). To the authors´ knowledge, no previous studies have evaluated the bacterial contamination of WSC used in mastectomies in small animals.

The objectives of this case series were to describe the use of WSC to administer postoperative local analgesia by the owners in dogs with mammary tumors managed with mastectomy, to identify complications associated with WSC and to determine the frequency of bacterial colonization of the catheters.

## Materials and methods

### Animal

The study protocol was approved by the Institutional Ethics Committee (ref.07/2017). Owner consent was obtained. Inclusion criteria included neutered female dogs with mammary tumors treated with a regional or radical unilateral mastectomy. Exclusion criteria included abnormalities on the preoperative physical examination or blood analysis; diagnoses of distant metastatic disease; or follow-up for less than 30 days.

Perioperative investigation and tumor staging consisted of complete physical examination, including palpation of each mammary gland and lymph nodes, hematology analysis, serum biochemical analysis, and thoracic radiographs. Radical mastectomy was performed when lesions > 3 cm or multiple nodules were present in the cranial and caudal glands.

### Surgical procedure and placement of WSC

Dogs were premedicated with 30 µg/kg acepromazine (10 mg/ml; Neosan®) and 0.3 mg/kg methadone (10 mg/ml; Semfortan®) intramuscularly. Anesthesia was induced with alfaxalone (10 mg/ml; Alfaxan®) and maintained with isoflurane vaporized in oxygen (IsoVet®). Intraoperative rescue analgesia was provided with fentanyl (2 µg/kg; Fentanest®) IV, if mean arterial pressure or HR increased more than 20% from its previous values within one minute. Meloxicam (0.2 mg/kg, IV, Metacam® 1.5 mg/ml) was administered after surgery. The surgical area was clipped and aseptically prepared. Prophylactic intravenous antibiotic (cefazolin 22 mg/kg; Cephacare®) was administered at the time of anesthetic induction and repeated every 120 min until the surgical procedure was completed.

Surgical procedures were performed by the same experienced surgeon according to a standardized surgical technique. An elliptical skin incision was made around the mammary glands with a minimum of a 2 cm margin from the lesion, and subcutaneous tissue was dissected to expose the muscle fascia. A new skin incision was created to introduce the WSC (MILA-International®), and the fenestrated part of the catheter was inserted into the subcutaneous space along the entire length of the surgical wound, so the WSC was in the deepest layer of the wound, running between the subcutaneous tissue and over the fascia. The size (DIFF 4, 7 and 9, with numbers indicating the length of perforated catheter in inches) and number of the catheters were selected based on the length of the wound. The catheter was secured to the skin with a purse-string and finger trap suture pattern at the catheter exit, and by two simple sutures at the securing device provided by the manufacturer in the sterile kit. The catheter tip was covered with a sterile injection cap.

The skin defect was closed in three layers with approximating sutures (over the catheter and not engaging it) followed by a subcuticular continuous pattern using 2 − 0 and 3 − 0 suture (Monosyn®) respectively. Alternatively, approximating sutures could be placed before introducing the catheter, which would be then passed under those sutures with the aid of a curved mosquito forceps. Simple interrupted sutures (3 − 0 nylon) were used in the skin with. The surgical wound and the catheter exit incision were covered with soft sterile wound dressings. Anesthesia time was defined as the time from the induction to extubation of the patient. Surgical time was defined as time from first incision to completion of skin closure. Hypothermia was defined as mild (36.7–37.7º C), moderate (35.5–36.7ºC), or severe (33.0-35.5ºC). Hypotension was defined as a mean arterial blood pressure less a than 60 mmHg and measured using an oscillometric non-invasive arterial blood pressures device and cuff that measured approximately 40% of limb circumference. All excised tissue underwent histopathological analysis, classified as benign or malignant using criteria previously described (Evans et al. 2021).

### Postoperative period

Bupivacaine (5 mg/ml; B.Braun®) was locally administered through the WSC at 1–2 mg/kg/6 h, aiming for 1 ml per 5 cm of incision length. If the minimum volume was not achieved with bupivacaine alone, sterile saline was added. Initial administration occurred just before extubation, with subsequent doses every 6 h until catheter removal. Methadone 0.3 mg/kg IV (10 mg/ml; Semfortan®) was administered every 4 h. The short form of the Glasgow Composite Measuring Pain Scale (CMPS-SF) was used to assess postoperative pain during hospitalisation. Two patients received rescue analgesia with methadone (0.1 mg/kg) IV and were reassessed 30 min later. An Elizabethan collar was placed, and its use was recommended until suture removal (12 days after surgery). Patients were discharged 24 h after surgery. Postoperative care included carprofen (Per Os (PO) 2 mg/kg/12 hours/5days; ) and tramadol (PO 3 mg/kg/12hours/3days; Tralieve®). No postoperative antibiotics were prescribed. Owners received pre-loaded single-dose syringes of bupivacaine at the calculated dose. They were instructed to store the syringes in a cool, sunlight-free place and administer the bupivacaine solution slowly through the injection cap every 6 h. The instructions included aseptic handling of the WSC injection cap using gloves, and injection of the bupivacaine taking 1–2 min to complete the injection. Additionally, owners administered the first dose of bupivacaine under direct veterinary supervision of the veterinary at the hospital prior to discharge.

The WSC was aseptically removed at 3 days postoperatively (first follow-up). Dogs were also re-checked on day 7 and 12 postoperatively. Postoperative complications were defined as any clinical sign or adverse event concerning the wound that deviated from the ideal course of wound healing and was associated with the surgical intervention, including WSC placement. Complications were classified based on the severity as mild (requires minor procedures such as wound drainage or antibiotic use), moderate (requires pharmacological treatment other than antibiotics), or severe (requires additional surgical procedures or organ failure is present) (Follette et al., 2020). Owners were instructed to monitor the visible skin around the wound (the wound itself being covered with sterile padding) for any swelling, redness, or discharge. Owners were instructed to monitor for any adverse event such swelling, redness, or discharge. Potential signs of pain were also discussed with the owners. SSI was determined based on the criteria previously described by the Centers for Disease Control and Prevention (Berríos-Torres et al. 2017).

### WSC processing and bacterial culture

Before removing the catheter, the skin around its exit was aseptically prepared. The WSC was removed and handled under sterile conditions. The portion of the WSC that had been under the skin was divided into three segments of approximately 10 cm. Each segment was placed into a separate sterile tube and sent to a microbiology laboratory in the same building. Once in the laboratory, the three catheter segments were further cut into smaller pieces. Each of those pieces was cut longitudinally, to open and fully expose the internal lumen, and was placed in a sterile tube. One ml of buffer solution (physiological saline, Sigma-Aldrich®) was subsequently added to each tube. Then, 100 µl from each tube was collected and mixed with the contents of the other tubes with samples from the same segment. Samples from each WSC were seeded onto 6 plates of Blood Agar (3 plates) and MacConkey (3 plates), 1 plate of each medium. The samples were incubated at 37ºC for 72 h. When bacterial growth occurred, colony forming units (CFU) were counted and the bacterial species identified. Samples with bacterial growth were considered positive.

### Statistical analysis

For continuous variables, the normality of the data distribution was assessed using the Shapiro test. Continuous normal data were presented as mean [standard deviation (SD)] and continuous non-normal data were presented as median, interquartile range (IQR), and minimum and maximum. Categorical variables were expressed as percentages. Excel (Microsoft®) was used for data collection. Statistical analyses were performed using Stata (Stata Corp®).

## Results

In total, 18 spayed female dogs underwent mastectomy during the study period. Seven patients were excluded due to abnormalities on the preoperative physical examination and blood analysis. Therefore, eleven spayed females of different breeds were included. Mean (SD) age was 9.2 (2.6) years, ranging 5–14 years, and median body weight was 9.0 kg (IQR 5,0–16,2 kg), range 6–38 kg. Six regional and five radical unilateral mastectomies were performed (Supplementary data).

Total anesthetic time ranged 90–180 min (90–170 min for partial mastectomy and 119–180 min for radical mastectomy) with a mean (SD) of 124.5 (33.7) min (106.7 [31.6] min for partial mastectomy and 145.80 [23.3] min for radical mastectomy). Total surgical time ranged from 45 to103 min (45–103 min for partial mastectomy and 70–100 min for radical mastectomy) with a mean (SD) of 74.6 (20.6) min (64.8 [21.5] min for partial mastectomy and 86.2 [13.1] min for radical mastectomy). Anesthetic complications were recorded in all patients and included hypothermia (*n* = 11) (1 mild, 5 moderate, 5 severe) and hypotension (*n* = 4). Hypothermia was treated with a warm air blanket during the surgery and the immediate postoperative period. Hypotension was treated by dopamine (2mcg/kg/min). A second dose of intraoperative antibiotics was administered to 5 patients. Twelve WSC were placed, including one DIFF 4 (9.1%), seven DIFF 7 (54.5%) and four DIFF 9 (36.4%). Two DIFF 7 WSCs were placed in one dog. No complications related with the placement of the WSC were identified.

According to the 11 owners, bupivacaine was correctly administered with no problems. All WSC were removed with no associated complications at the first follow-up. No complications related with the WSC were identified by the owners or veterinary practitioners in the subsequent follow-ups. Bacteriological culture was negative in all the samples from the 12 WSC. Benign conditions (lobular hyperplasia) were diagnosed in 5 dogs (45.5%), and malignant condition were described in 6 dogs (54.5%), all of which were diagnosed as malignant carcinoma.

## Discussion

In this case series, no complications were associated with the postoperative use of 12 WSC in 11 dogs with mammary tumors treated surgically. Additionally, no bacterial growth was identified in the culture of any of the samples from the 12 WSC. To the authors’ knowledge, this is the largest published study reporting the use of WSC following mastectomy.

In this study, all the WSC were successfully managed by the owners at home following patient discharge. No previous report described the use of WSC by owners. A retrospective study (Lu and Wright 2023) compared complications for closed suction subcutaneous drains managed either in the hospital or at home by owners. Home management showed a higher risk of minor complications. However, owners successfully addressed these issues without reported complications, and correct drug administration occurred. Another benefit was a significantly shorter hospitalization compared to hospital management. Results suggest home management of subcutaneous drains post-hospital discharge as a safe, viable option to reduce hospitalization duration and related consequences, such as nosocomial infection, expense, and stress. Findings of our study suggest that hospital discharge with a subcutaneous WSC, could be a safe and feasible option.

Reported complications associated with WSC include disconnection of the catheter, premature catheter removal by the patient, catheter occlusion, lidocaine neurotoxicity, seroma, and surgical site infection (Radlinsky et al. 2005; Abelson et al. 2009). Other reported complications associated with different closed drains are increased wound drainage, delayed wound healing and increased surgical site infection rate (Lu and Wright 2023). In contrast, no complications associated with WSC were identified in this study. As previously mentioned, the use of wound drains has also been identified as a risk factor associated with SSI in some studies (Lu and Wright 2023) but not by others (Espinel-Rupérez et al. 2019). These agree with our results, because despite the management of the WSC at home by the owners and the absence of postoperative antibiotics, no SSI was identified in this study. Seroma formation has also been described as another possible complication associated to WSC and wound drains in veterinary studies (Wolfe et al. 2006; Abelson et al. 2009) but not in others studies (Radlinsky et al. 2005), similar to our case series. The absence of seroma formation in our study could be secondary to the low number of cases. However, the use of gentle surgical dissection of the mammary tissue following Halsted principles, and the use of postoperative dressings, may have contributed to prevention of this complication.

There is scarce information in veterinary medicine regarding bacterial colonization associated to WSC or wound drains. A study in dogs that underwent total ear canal ablation, identified only one positive bacterial culture *(Enterococcus faecalis)* from the eight WSC analysed (Radlinsky et al. 2005). Another study including 80 closed drains placed in clean surgeries in 77 dogs described an incidence of infection of 15.6% (Bristow et al. 2015). In contrast, all the microbiologic cultures from the 12 WSC were negative in our study. Bacterial cultures from wound drains have been widely reported with an incidence of positive cultures ranging from 6.2 to 30% (Degnim et al. 2014). An antimicrobial effect has been previously associated with the administration of bupivacaine due to disruption of microbial cell membrane permeability, and subsequent bacteria cell lysis (Razavi and Fazly Bazzaz 2019). Therefore, this antimicrobial effect could have contributed to the low rate of bacterial contamination.

The main limitations of the study are the small number of patients included and the lack of a control group. Additionally, only healthy spayed dogs with no other conditions apart from mammary tumors were included in the study, which is not the only clinical presentation of cases, since females undergoing mastectomy may require an ovariohysterectomy and have co-morbidities (Evans et al. 2021). Despite its limitations, this is the first description of potential complications and bacterial contamination of WSC used to administer postoperative local analgesia by owners of dogs with mammary tumors treated with mastectomy.

## Conclusions

Findings of this report suggest that the use of WSC following mastectomy in dogs is associated with a low number of complications and no bacterial colonization. Future randomized studies involving a larger number of dogs are required.

### Electronic supplementary material

Below is the link to the electronic supplementary material.

Supplementary Material 1

## Data Availability

No datasets were generated or analysed during the current study.
