# Supplementary material for: Description of postoperative complications and bacterial contamination of wound soaker catheters used to administer postoperative local analgesia after mastectomy in 11 dogs: case series
Source: Vet Res Commun. 2024 Apr 24;48(4):2707–12. doi: 10.1007/s11259-024-10377-1 (PMC11315717; doi:10.1007/s11259-024-10377-1)
Supplement: Supplementary file 1 — Supplementary Material 1 [file 11259_2024_10377_MOESM1_ESM.docx]

| **TABLE 1. Supplementary data. Summary data collected from the patients.** | | | | | | | | | |
| --- | --- | --- | --- | --- | --- | --- | --- | --- | --- |
| **Case** | **Number of WSC used** | **Type of mastectomy** | **Breed** | **Weight (kg)** | **Age (months)** | **Previous disease** | **Current treatment** | **Previous surgery (apart from ohe)** | **Duration of anesthesia (min)** |
| 1 | 1 | Radical L1-L5 | Cairn Terrier | 5.7 | 108 | no | no | no | 119 |
| 2 | 1 | Regional L1-L3 | Cross-breed | 9.0 | 96 | no | no | no | 90 |
| 3 | 2 | Radical L1-L5 | Cocker Spaniel | 5 | 96 | no | no | no | 180 |
| 4 | 1 | Regional R3-R5 | Jack Russel | 9.8 | 60 | no | no | no | 90 |
| 5 | 1 | Regional L3-L5 | Cross-breed | 7.2 | 120 | no | no | no | 90 |
| 6 | 1 | Regional L3-L5 | Siberian Husky | 16.2 | 120 | no | no | no | 105 |
| 7 | 1 | Regional L3-L5 | Cross-breed | 26.5 | 168 | no | no | no | 170 |
| 8 | 1 | Radical R1-R5 | Yorshire Terrier | 2.4 | 132 | no | no | no | 130 |
| 9 | 1 | Radical L1-L5 | Yorshire Terrier | 2.8 | 120 | no | no | no | 150 |
| 10 | 1 | Regional L3-L5 | Cross-breed | 38.0 | 60 | no | no | no | 95 |
| 11 | 1 | Radical R1-R5 | Cross-breed | 11.1 | 132 | no | no | no | 150  (continues) |

| **TABLE 1. Supplementary data. Summary data collected from the patients.** | | | | | | | | | | |
| --- | --- | --- | --- | --- | --- | --- | --- | --- | --- | --- |
| **Case** | **Presence of hypothermia during anesthesia** | **Presence of hypotension during anesthesia** | **Type of WSC** | **Blood agar result** | **MacConkey agar result** | **Duration of surgery (min)** | **Second dose of prophylactic antibiotic** | **Postoperative complication** | **Malignant tumour** | **Type of malignant tumour** |
| 1 | moderate | no | DIFF 9 | Negative | Negative | 70 | no | no | yes | Carcinoma |
| 2 | mild | no | DIFF 4 | Negative | Negative | 50 | no | no | no | _ |
| 3 | moderate | no | DIFF 7 | Negative | Negative | 90 | yes | no | yes | Carcinoma |
| 4 | severe | no | DIFF 7 | Negative | Negative | 70 | no | no | no | _ |
| 5 | moderate | no | DIFF 7 | Negative | Negative | 45 | no | no | no | _ |
| 6 | severe | no | DIFF 9 | Negative | Negative | 51 | no | no | yes | Carcinoma |
| 7 | moderate | no | DIFF 7 | Negative | Negative | 103 | yes | no | no | _ |
| 8 | severe | yes | DIFF 7 | Negative | Negative | 75 | yes | no | no | _ |
| 9 | severe | yes | DIFF 9 | Negative | Negative | 96 | yes | no | yes | Carcinoma |
| 10 | moderate | yes | DIFF 7 | Negative | Negative | 70 | no | no | yes | Carcinoma |
| 11 | severe | yes | DIFF 9 | Negative | Negative | 100 | yes | no | yes | Carcinoma |
